# Generation of immunocompetent syngeneic allograft mouse models for pediatric diffuse midline glioma

**DOI:** 10.1093/noajnl/vdac079

**Published:** 2022-05-24

**Authors:** Aimée du Chatinier, Michaël H Meel, Arvid I Das, Dennis S Metselaar, Piotr Waranecki, Marianna Bugiani, Marjolein Breur, Erin F Simonds, Edbert D Lu, William A Weiss, Juan J Garcia Vallejo, Eelco W Hoving, Timothy N Phoenix, Esther Hulleman

**Affiliations:** Princess Máxima Center for Pediatric Oncology, Utrecht, the Netherlands; Princess Máxima Center for Pediatric Oncology, Utrecht, the Netherlands; Princess Máxima Center for Pediatric Oncology, Utrecht, the Netherlands; Princess Máxima Center for Pediatric Oncology, Utrecht, the Netherlands; Princess Máxima Center for Pediatric Oncology, Utrecht, the Netherlands; Department of Pathology, Amsterdam University Medical Centers, Amsterdam, the Netherlands; Department of Pathology, Amsterdam University Medical Centers, Amsterdam, the Netherlands; Departments of Neurology, Neurological Surgery, and Pediatrics, Helen Diller Family Comprehensive Cancer Center, University of California San Francisco, San Francisco, California, USA; Departments of Neurology, Neurological Surgery, and Pediatrics, Helen Diller Family Comprehensive Cancer Center, University of California San Francisco, San Francisco, California, USA; Departments of Neurology, Neurological Surgery, and Pediatrics, Helen Diller Family Comprehensive Cancer Center, University of California San Francisco, San Francisco, California, USA; Department of Molecular Cell Biology and Immunology, Amsterdam University Medical Centers, Amsterdam, the Netherlands; Princess Máxima Center for Pediatric Oncology, Utrecht, the Netherlands; Division of Pharmaceutical Sciences, College of Pharmacy, University of Cincinnati/Research in Patient Services, Cincinnati Children’s Hospital Medical Center, Cincinnati, Ohio, USA; Princess Máxima Center for Pediatric Oncology, Utrecht, the Netherlands

**Keywords:** DMG, DIPG, immunotherapy | syngeneic allograft, tumor microenvironment

## Abstract

**Background:**

Diffuse midline gliomas (DMG) are highly malignant incurable pediatric brain tumors. A lack of effective treatment options highlights the need to investigate novel therapeutic strategies. This includes the use of immunotherapy, which has shown promise in other hard-to-treat tumors. To facilitate preclinical immunotherapeutic research, immunocompetent mouse models that accurately reflect the unique genetic, anatomical, and histological features of DMG patients are warranted.

**Methods:**

We established cell cultures from primary DMG mouse models (C57BL/6) that were generated by brainstem targeted intra-uterine electroporation (IUE). We subsequently created allograft DMG mouse models by orthotopically implanting these tumor cells into syngeneic mice. Immunohistochemistry and -fluorescence, mass cytometry, and cell-viability assays were then used to verify that these murine tumors recapitulated human DMG.

**Results:**

We generated three genetically distinct allograft models representing histone 3 wildtype (H3^WT^) and K27M-mutant DMG (H3.3^K27M^ and H3.1^K27M^). These allograft models recapitulated the histopathologic phenotype of their human counterparts, including their diffuse infiltrative growth and expression of DMG-associated antigens. These murine pontine tumors also exhibited an immune microenvironment similar to human DMG, characterized by considerable myeloid cell infiltration and a paucity of T-lymphocytes and NK cells. Finally, we show that these murine DMG cells display similar sensitivity to histone deacetylase (HDAC) inhibition as patient-derived DMG cells.

**Conclusions:**

We created and validated an accessible method to generate immunocompetent allograft models reflecting different subtypes of DMG. These models adequately recapitulated the histopathology, immune microenvironment, and therapeutic response of human DMG, providing useful tools for future preclinical studies.

**Key Points**

- Allografting IUE-transformed murine tumor cells generates immunocompetent DMG models.
- DMG allografts recapitulate histopathologic and immunologic features of human DMG.

**Importance of the Study**

Our study describes the generation of immunocompetent DMG mouse models that enable preclinical research on the potential—and risk—of immunotherapy for this devastating disease. Applying a two-step system, we first established primary tumor cell lines from murine DMG tumors that were generated by brainstem-targeted intra-uterine electroporation. This method enabled us to manipulate expression within the intact developing brainstem, thereby inducing spontaneous tumor formation in a spatially and temporally controlled manner. We then created allograft DMG mouse models by orthotopically implanting these primary tumor cells in syngeneic mice. Herewith, we provide an allograft tool that is more stable in phenotype, better suitable for large-scale therapeutic studies and easily accessible. Importantly, we show that these murine DMG models accurately recapitulate the growth pattern, morphology, and immune microenvironment of human DMG. As such, these models allow for novel, urgently needed therapies to be tested in a controlled environment that better reflects human disease.

Diffuse midline gliomas (DMG) are universally fatal brain tumors that arise in midline structures of the CNS, such as the thalamus and brainstem.^1^ Their localization in critical brain structures and aggressive nature results in a devastating prognosis, with an median overall survival of 11 months after diagnosis and less than 1% survival past 5 years.^2^ Recent international collaborations have uncovered several oncogenic mutations in this disease, with the most frequent genetic alterations resulting in a lysine-to-methionine substitution at amino acid 27 of histone 3 proteins (mainly H3F3A^K27M^ or HIST1H3B^K27M^).^3,4^ Other recurrent mutations involve inactivation of p53 and amplification or constitutive activation of PDGFRA and/or ACVR1.^3,4^ Despite advances in understanding the molecular basis of DMG, there is an ongoing challenge within the field of pediatric neuro-oncology to develop effective treatments.^5,6^ To this day, the current standard of care—consisting primarily of radiotherapy—merely provides symptom relief and a delay in tumor progression, but is never curative.^2^

One of the major breakthroughs in cancer research over the last two decades has been the development of immunotherapy.^7,8^ Although immunotherapy has significantly improved survival rates for some cancer types, the absence of immune cell infiltration has considerably hampered the success for so-called immunologically “cold” tumors.^9^ It is now increasingly accepted that the composition of the tumor immune microenvironment (TIME) impacts responsiveness to immunotherapy and therefore acts as a pivotal factor for effective immunotherapeutic strategies.^10,11^ Research on the DMG immune landscape is limited, but initial reports show that these tumors are characterized by a non-inflammatory TIME that consists largely of tolerogenic myeloid cells and very few lymphoid cells.^12,13^ Case reports from preliminary clinical studies show that immunotherapy can be applied safely to pediatric glioma patients.^14,15^ However, it has yet to be established whether these treatments can lead to improvements in their outcome or long-term remissions, especially considering the immunologically cold phenotype of these tumors.

A major hurdle that prevents preclinical immunological research in DMG is the absence of representative animal models with an intact immune system. The most frequently used *in vivo* models for DMG studies are established by xenografting biopsy or autopsy tissue in immunodeficient mice, i.e., patient-derived orthotopic xenografts (PDOX).^16^ Consequently, the absence of an intact immune system generally precludes the use of these models for immunological studies. Until recently, we relied on the use of carcinogen-induced or genetically engineered glioma models, as these are intrinsically immunocompetent and can be allografted in syngeneic immunocompetent animals without immediate immune rejection.^17,18^ However, these models do not accurately reflect the molecular background of DMG, lack flexibility due to extensive breeding schemes, or develop tumors at an inappropriate location in the brain.^19–22^ Thus, there is a dire need to develop representative immunocompetent DMG models that are amendable to study the potential—and risk—of immunotherapy in the different DMG subclasses.

In this study, we describe a platform to generate immunocompetent DMG mouse models representing three genetically distinct DMG subclasses. We first established tumor cell lines from primary DMG mouse models that were generated by introducing DMG subtype-specific mutations into the embryonic brainstem by intra-uterine electroporation (IUE). We subsequently implanted these primary murine tumor cells orthotopically as allografts in syngeneic mice, resulting in the rapid generation of secondary brain tumors that reflect the histopathological characteristics of human DMG. Furthermore, we show that the TIME of these tumors resembles the immune-cold microenvironment observed in patient material. Herewith, we provide a valuable and accessible tool to generate representative DMG mouse models with an immunocompetent and controlled genetic background.

## Materials and Methods

### Patient Material

Tumor tissue was obtained through autopsy of DMG patients at the Amsterdam University Medical Center (Amsterdam, the Netherlands), as described previously.^23^ All patient material was collected according to approved institutional ethical guidelines and in accordance with the declaration of Helsinki.

### Cell Cultures and Culture Conditions

The murine DMG cell lines UC-BL6-D1, UC-BL6-D3, UC-BL6-B1, UC-BL6-B7, UC-BL6-C1, UC-BL6-C2, and UC-BL6-C7 (Supplementary Table 1) were derived from primary murine tumors generated by brainstem targeted IUE of PiggyBac DNA plasmids, as described previously^24^ and briefly outlined in Supplementary Material.

The human primary cell line VUMC-DIPG-10 (H3^WT^) was established from autopsy material at the Amsterdam University Medical Center (Amsterdam, the Netherlands), as described previously.^25^ HSJD-DIPG-07 (H3.3^K27M^) was a kind gift from Dr. Montero Carcaboso (Hospital San Joan de Déu, Barcelona, Spain), JHH-DIPG-01 (H3.3^K27M^) from Dr. Raabe (John Hopkins Hospital, Baltimore, MD), and SU-DIPG-IV (H3.1^K27M^), and SU-DIPG-XXI (H3.1^K27M^) from Dr. Monje (Stanford University, Stanford, CA).

Murine and human cells were cultured as neurospheres as described previously.^25^ All human cell lines were authenticated by short tandem-repeat profiling to ensure cell identity. All cell lines were routinely subjected to mycoplasma testing and only used for experiments when confirmed negative.

### Cell Viability Assays

Cell viability assays were performed as described previously using Panobinostat (LBH 589) (Axon Medchem).^26^

### In Vivo Studies

All animal experiments were performed in accordance with national and institutional regulations and were approved by the Institutional Animal Care and Use Committees of the University of Cincinnati and the Vrije Universiteit Amsterdam. Supportive care was provided as indicated by these guidelines. Animals were provided food and water *ad libitum* for the entire duration of the experiments. A detailed description of the *in vivo* experiments is reported in Supplementary Material.

### Immunohistochemistry (IHC) and Immunofluorescence (IF)

Patient and murine tissue samples were immunostained as described previously^23,27^ with antibodies against Ki67, H3K27M, Iba1, CD3, IL13Rα2, Gfap, Olig2, NKp46, CD45, and CD57, as detailed in Supplementary Material.

### Flow-Cytometric Analysis

GD2 immunophenotyping was performed by incubating cells for 30 min at 4°C with PE-conjugated anti-GD2 antibody (clone 14.G2a) (1:100; #562100, BD Biosciences) in staining buffer (0.2% BSA in PBS) in the dark, as described previously.^28^ Flow cytometric analysis was performed on a CytoFLEX S Flow Cytometer (Beckman Coulter).

### Cytometry by Time of Flight (CyTOF)

Experimental details for CyTOF procedures, including data acquisition and analysis, are outlined in Supplementary Material.

### Statistics

All in vitro data are represented as averages (mean ± s.d.) from at least three technical replicates. In vitro dose response curves were fitted with the log(inhibitor) vs. response–variable slope (four parameters) curve to determine the IC50. In vivo Kaplan–Meier curves were compared using the Tarone–Ware test. Analyses were performed using GraphPad Prism (version 8.0.2), IBM SPSS Statistics (version 27), or Microsoft Excel (version 14.7.2). *P*-values below .05 were considered statistically significant.

## Results

### Generation and Histopathologic Validation of Immunocompetent Murine DMG Models

**Figure 1.**
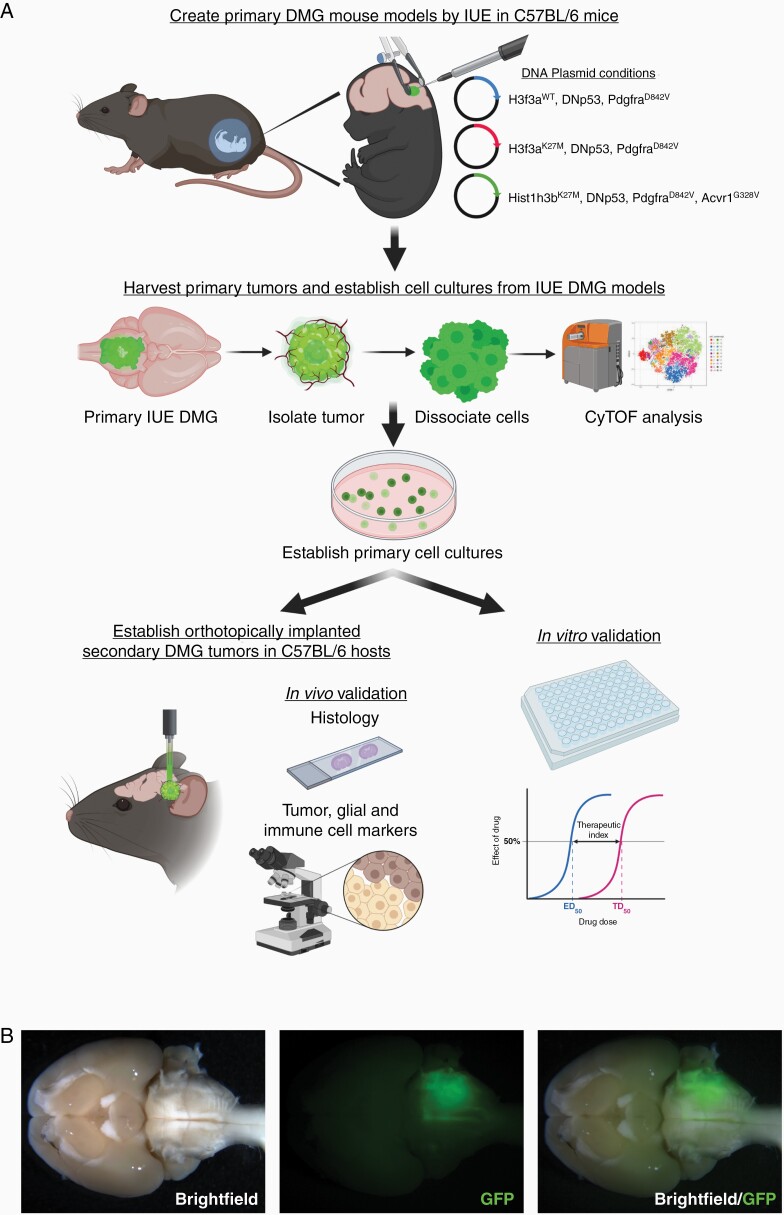
Generation of immunocompetent DMG mouse models. (A) Graphical overview. Figure was made with BioRender. (B) Representative ventral whole brain brightfield and GFP images demonstrating the location of a GFP-positive H3.3^K27M^ tumor generated by intra-uterine electroporation (IUE). Images of H3^WT^ and H3.1^K27M^ models are shown in Supplementary Figure S1.

We created native forming brainstem DMG mouse models by performing brainstem targeted IUE in C57BL/6 embryos with PiggyBac DNA plasmids, as described previously (Figure 1A and B, Supplementary Figure S1).^24^ Three genetically distinct IUE models were produced, representing histone 3 wildtype (H3^WT^), histone 3.3 mutant (H3.3^K27M^), and histone 3.1 mutant (H3.1^K27M^) DMG. All three models expressed constitutively active Pdgfra (D842V mutant) and dominant negative Trp53 (DNp53) but varied by their expression of histone 3 mutations (H3f3a^WT^, H3f3a^K27M^, or Hist1h3b,^K27M^ respectively). Additionally, H3.1^K27M^ models also expressed mutant Acvr1 (G328V), which is enriched for in H3.1^K27M^-mutant DMG.^3,4^ Following birth, successfully electroporated offspring was monitored for development of neurologic symptoms related to tumor burden, at which time whole brains were harvested (Figure 1A and B, Supplementary Figure S1). Similar to prior studies, the addition of H3f3a^K27M^ or Hist1h3b^K27M^ was associated with accelerated tumor development (median survival: H3^WT^ = 77 days; H3.3^K27M^ = 39 days; H3.1^K27M^ = 44 days; *P* = 0.002) (Supplementary Figure S1A).^24^ Subsequently, primary neurosphere cultures were established from individual GFP-positive tumor tissue that was microdissected and then dissociated into single cell suspensions (Figure 1A and B  Supplementary Figure S1B). We generated 9 unique cell lines (3 H3^WT^, 3 H3.3^K27M^, and 3 H3.1^K27M^), 7 of which went on to be further tested in these studies (Supplementary Table 1). Each cell line expressed fluorescent and bioluminescent markers from IRES-linked elements built into DNp53 (IRES-Luciferase) or Pdgfra/histone (IRES-GFP) plasmids. Established murine IUE DMG neurosphere cultures were then tested for their ability to form secondary tumors in syngeneic hosts (Figure 1A). Orthotopic implantation of IUE DMG cells into the brainstem (pons) of C57BL/6 mice resulted in rapid engraftment across tumor cell genotypes (Supplementary Figure S2). Notably, in contrast to the primary IUE DMG models, histone mutation status did not enhance secondary tumor formation (median survival: H3^WT^ = 19 days; H3.3^K27M^ = 49 days; H3.1^K27M^= 36 days; *P* = 0.073) (Supplementary Figure S2).

**Figure 2.**
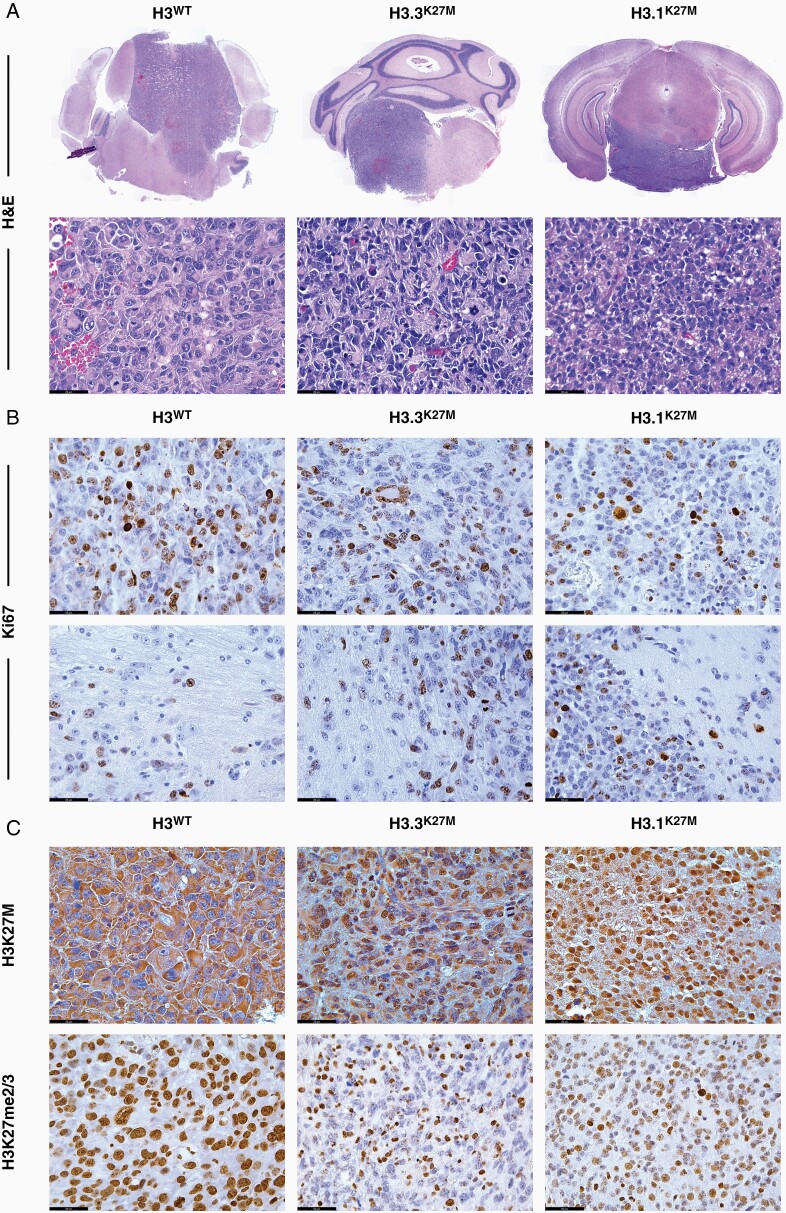
Histopathological validation of allograft DMG mouse models. (A) Representative coronal sections (top panels) and high magnification (400×, bottom panels) images of H&E stained DMG allograft tumors across genetic conditions. (B) Representative images (400×) of Ki67 immunohistochemical staining (in brown) of DMG allograft tumor sections showing the tumor core (top panels) and diffuse, infiltrative growth areas (bottom panels). (C) Representative images (400×) of immunohistochemical staining (in brown) for nuclear mutant histone 3 protein (H3K27M, top panels) and histone 3 lysine 27 di- and trimethylation (H3K27me2/3, bottom panels) of DMG allograft tumor sections. Scale bars = 50 μm.

To validate that the allograft tumor models resemble human DMG, we analyzed their morphology and growth pattern by IHC and IF. All tumors showed histopathological characteristics of human DMG, including diffuse infiltrative growth through the midline of the brain, nuclear atypia, cellular pleomorphism, and increased and abnormal mitotic activity (Figure 2A and B, Supplementary Figures S3–S5). In addition to growth at the primary site of implantation, we observed tumor cell spread into the cerebellum and alongside the ventricles into the cerebral cortex, corresponding to the diffuse nature of DMG and invasive patterns found in DMG PDOX implant models (Supplementary Figures S3–S5).^23,29^ Occasionally, these tumors displayed a giant cell component, a characteristic previously described in human DMG cases, as well as in primary IUE mouse glioma models (Supplementary Figure S3).^24^ H3^WT^ allograft tumors were classified as grade IV, demonstrating a high cellular density, epithelioid cellular morphology, and regions of necrosis, but no overt vascular proliferation (Figure 2A, Supplementary Figure S3). Both H3.3^K27M^ and H3.1^K27M^ allograft tumors were characterized as grade III, with a moderate to high cellular density, glial cellular morphology, but no areas of necrosis or vascular proliferation (Figure 2A, Supplementary Figures S4 and S5). Ki67-positive proliferative tumor cells were present in all conditions, although overall proliferation rate varied throughout the tumor (Figure 2B, Supplementary Figures S3–S5). To confirm the maintenance of histone 3 status from primary IUE DMG tumors, we stained for the mutant histone 3 protein (H3K27M) and loss of histone 3 lysine 27 di- and trimethylation (H3K27me2/3), which is a hallmark of H3.3^K27M^ and H3.1^K27M^ tumors.^23^ Immunohistochemical analysis demonstrated distinct nuclear immunopositivity for the mutant histone 3 protein specific to H3.3^K27M^ and H3.1^K27M^ allograft tumors, which also corresponded to loss of H3K27me2/3 immunoreactivity (Figure 2C, Supplementary Figure S4). H3^WT^ allograft tumors also contained H3K27me2/3 immunonegative areas, corresponding to what has been observed for H3^WT^ DMG patients (Supplementary Figure S3).^23^ All allograft models exhibited marked nuclear immunopositivity for oligodendrocyte transcription factor 2 (Olig2), a glial-restricted progenitor cell marker usually expressed by DMG cells (Supplementary Figure S6A).^23^ Expression of glial fibrillary acidic protein (Gfap) appeared restricted mainly to pre-existing astrocytes in each allograft tumor model, corresponding to previous IUE-generated and other murine DMG tumors (Supplementary Figure S6B).^24,29^ Taken together, we demonstrate that IUE DMG cell lines can be successfully used to generate orthotopic syngeneic allograft models that recapitulate the histopathologic phenotypes of human DMG.

### Murine DMG Tumors Exhibit an Immune Microenvironment Similar to Human DMG

**Figure 3.**
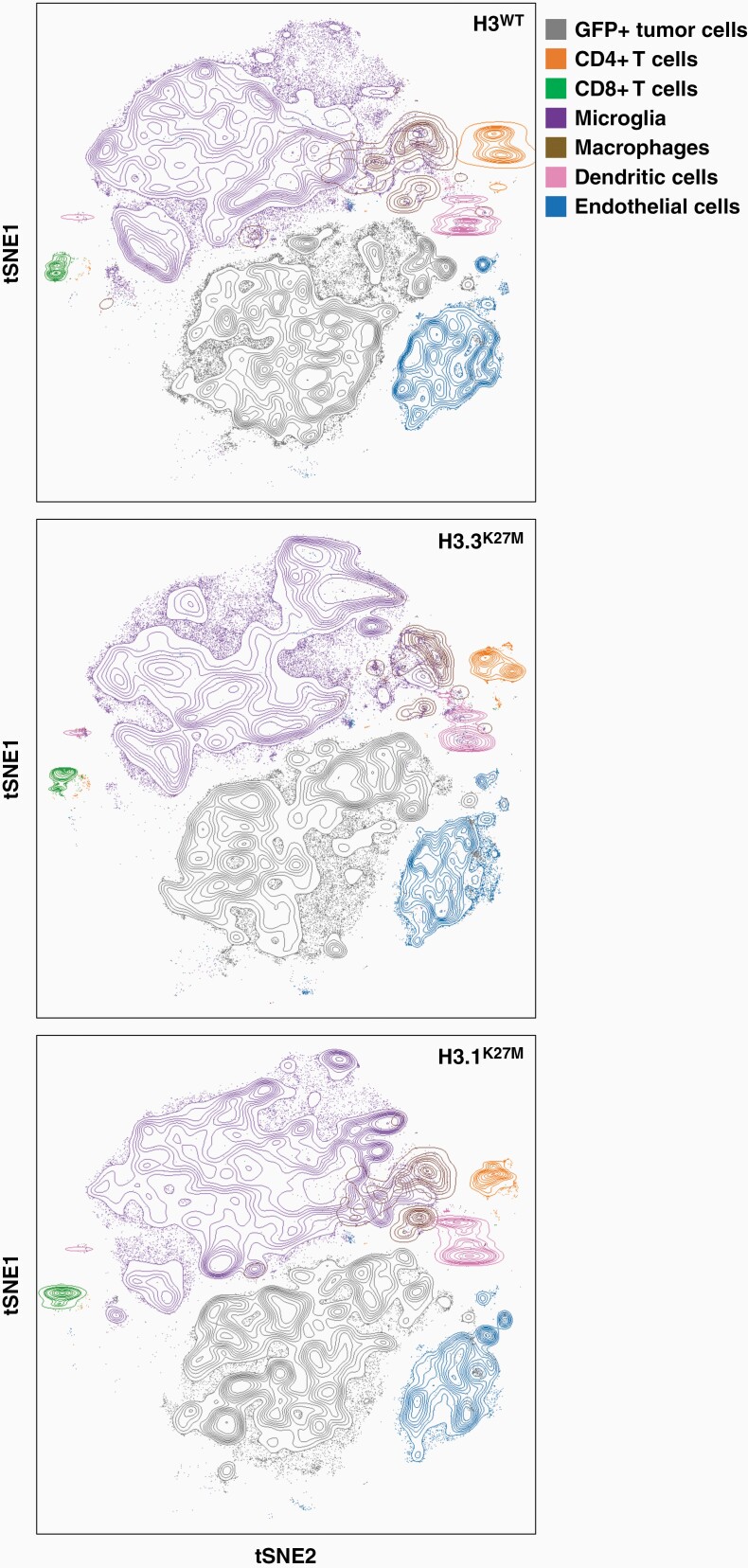
Characterization of the TIME in IUE DMG mouse models. T-distributed Stochastic Neighbor Embedding (optSNE) clustering of the TIME landscape of whole primary IUE brains across genetic conditions (CyTOF mass cytometry). Indicated cell populations are organized spatially by similarity and distinguished by color.

**Figure 4.**
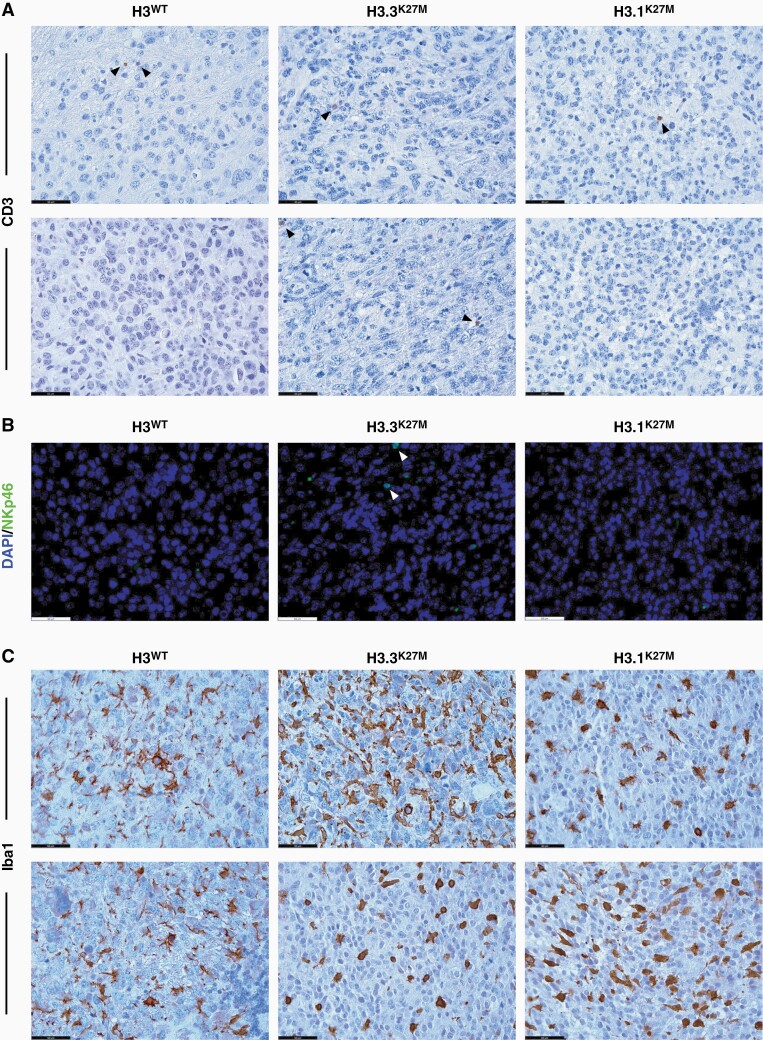
Characterization of the TIME in allograft DMG mouse models. (A) Representative images (400×) of CD3 immunohistochemical staining (in brown; marker for T-lymphocytes) of DMG allograft tumors across genetic conditions. Black arrowheads point to the sparsely present CD3-positive cells. (B) Representative immunofluorescent (400×) images of DMG allograft tumor sections co-stained for DAPI (blue) and NKp46 (red; marker for NK cells). White arrowheads point to the sparsely present NKp46-positive cells. (C) Representative images (400×) of Iba1 immunohistochemical staining (in brown; marker for microglia and macrophages) of DMG allograft tumor sections, showing the intra-tumoral heterogeneity with respect to microglia/macrophage density and phenotype. Scale bars = 50 μm.

To verify the immune competence of these mouse models, we first characterized the TIME of the primary IUE tumors by performing CyTOF mass cytometry on extracted whole tumor tissue, as described previously.^30^ Analysis of gated immune cell populations demonstrated that both histone wildtype and mutant IUE tumors consisted predominantly of myeloid cells, specifically microglia and macrophages, and very few lymphocytes, corresponding to what has previously been observed in human DMG autopsy tissue (Figure 3, Supplementary Figure S7).^12,13^ We subsequently analyzed the TIME in our secondary allograft models by staining for these immune cell populations by IHC and IF and comparing these to analogous human DMG tissues. While CD3-positive T-lymphocytes could sporadically be observed around hemorrhagic areas (Supplementary Figure S3B), little, if any, lymphocyte infiltration was observed throughout the tumor parenchyma for both murine allograft sections and human DMG tissues, irrespective of histone 3 mutational status (Figure 4A, Supplementary Figure S8A). Staining for NKp46, an NK cell-activating receptor, also demonstrated virtually no immunopositivity of NK cells in the murine allograft material, except for very few NKp46-positive cells observed in H3.3^K27M^ tumors (Figure 4B). Similarly, co-staining for CD45, a general marker for inflammatory cells, and CD57, a maturation marker for NK cells, in human autopsy tumor samples did not result in positive identification of NK cells within either of these tumors (Supplementary Figure S8B). These results demonstrate the predominant absence of T-lymphocytes and NK cells in the murine allograft models and human DMG tissues. In contrast, we observed a significant number of Iba1-positive microglia and macrophages in both murine allograft and human tumor tissue samples, although marked intra-tumoral heterogeneity was observed with respect to their cellular density and phenotype within the tumor parenchyma (Figure 4C, Supplementary Figure S8C). We noticed the presence of both microglia with branching processes and a small cellular body, and microglia with more retracted processes and enlarged cell bodies throughout the murine and human tumors, which suggests the presence of both ramified and reactive microglia, respectively (Figure 4C, Supplementary Figure S8C).

To verify that these immunocompetent DMG mouse models can be employed for the development of immunotherapies, we analyzed the expression of the disialoganglioside GD2 and Interleukin-13 receptor subunit alpha-2 (IL13Rα2), two antigens highly expressed in DMG tissues and identified as promising targets for immunotherapy in DMG.^28,31–34^ For Il13rα2, we could confirm expression in both histone 3 wildtype and K27M-mutant DMG tissues, although a slightly lower and more heterogenous expression was observed for histone 3 wildtype tumors (Supplementary Figure 9A). For GD2, flow cytometry analysis indicated that this antigen is also expressed in our murine DMG cells, irrespective of their histone mutational status (Supplementary Figure 9B). Altogether, these results show that our primary IUE and secondary syngeneic allograft murine models exhibit an immune microenvironment similar to human DMG and can be used to evaluate state-of-the-art immunotherapies.^12,13^

### Therapeutic Sensitivity of Murine DMG Tumors Is Equivalent to Patient-Derived DMG Cells

**Figure 5.**
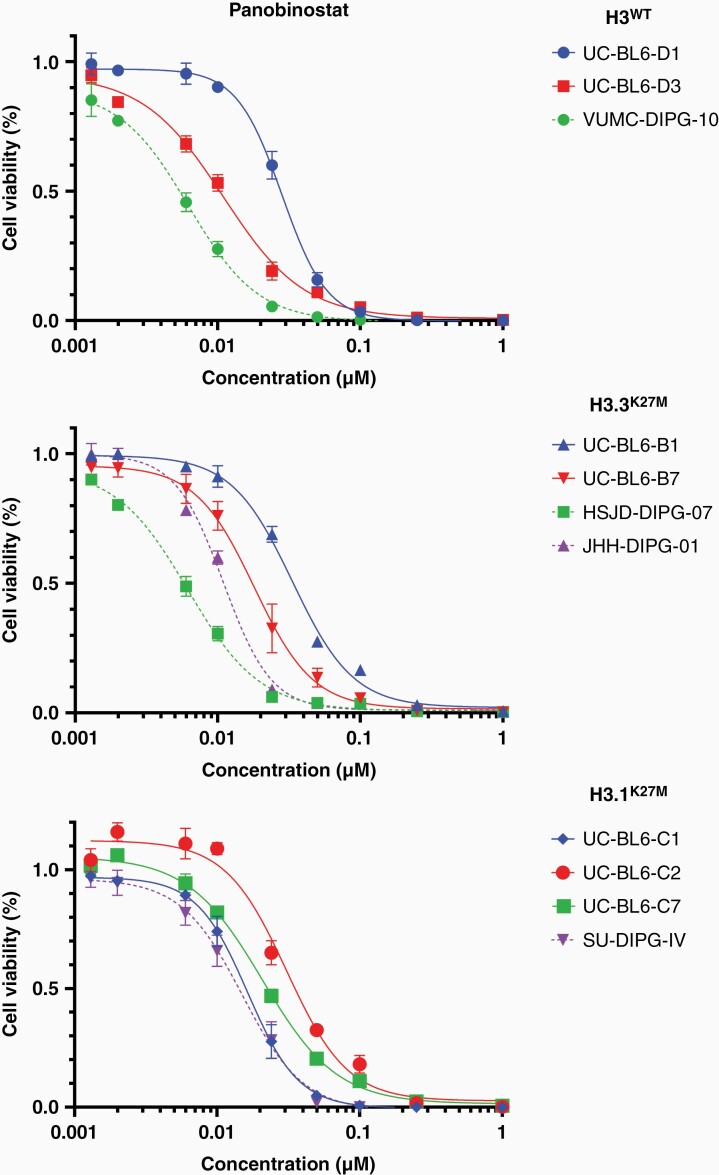
Therapeutic sensitivity of murine DMG tumor cells compared to patient-derived DMG cells. Dose–response curves representing cell viability of DMG cell lines generated by brainstem targeted IUE (solid lines) and analogous patient-derived DMG cell lines (dashed lines) across genetic conditions after 96-h treatment with Panobinostat. Data are represented as percentage viability compared to vehicle-treated controls, average ± s.d. (*n* = 3).

Preclinical models that closely recapitulate patients’ response to treatments are crucial to assess the efficacy of compounds prior to clinical applications. To validate the predictive value of our murine DMG tumors, we set out to investigate a small molecule inhibitor that has been tested preclinically and is currently in clinical trials for DMG. For this purpose, we chose the histone deacetylase (HDAC) inhibitor Panobinostat, a drug that has previously been identified as a potential therapeutic agent for DMG, and is currently in a phase I clinical trial for children with recurrent disease (NCT02717455).^35^ Furthermore, recent reports indicate that HDAC inhibitors may increase the efficacy of immune checkpoint inhibition in several types of cancer, even in the absence of an inflammatory microenvironment, indicating that the preclinical investigation of this compound in an immunocompetent setting is highly relevant for DMG.^36–39^ We measured treatment sensitivity of our IUE-generated murine DMG cells in vitro by exposing them to various concentrations of Panobinostat for 96h and measuring cell viability. We observed that sensitivity to Panobinostat corresponded to that observed for analogous patient-derived DMG cell lines, with IC50 concentrations between 10 and 100 nmol/L (Figure 5). Furthermore, sensitivity to Panobinostat was in line with previous publications using patient-derived DMG models, suggesting that our murine DMG models are suitable surrogates for testing therapeutic efficacy.^35,40^

## Discussion

DMG are highly aggressive and lethal pediatric brain tumors for which effective therapeutic options remain scarce. To evaluate novel treatments and advance research on DMG biology the generation of preclinical DMG modeling systems is imperative. Although in vitro experiments provide critical insights about the cellular and molecular features of brain tumors, limitations include the inability to adequately model invasion, angiogenesis, metastasis, and the influence of the tumor microenvironment on treatment response.^10^ Animal models that accurately recapitulate these processes are critical to evaluate therapeutic strategies prior to clinical testing.

Currently, the most frequently used in vivo models for DMG studies are established by xenografting biopsy or autopsy tissue in immunodeficient mice (i.e., PDOX).^16,18^ While these models can reproduce the natural context of human disease, a major limitation of the use of immunocompromised mice is that the immune system of these mice is (partially) absent to ensure successful tumor engraftment, thereby disrupting normal development of the TIME.^16^ Consequently, current PDOX models cannot be used to accurately study the TIME or therapeutic strategies that interact with the immune system.^16^ One solution to this problem is to use humanized xenograft hosts, in which the peripheral blood or bone marrow of the patient is co-engrafted with the tumor material into an immunodeficient mouse strain.^16^ Although this may be a promising strategy for future immunotherapeutic studies, no humanized xenograft models for pediatric brain tumors have yet been described, which may be ascribed to ethical restrains, extremely high costs, and complicated procedures including neo-adjuvant chemo- and radiotherapy to enable engraftment of human cells in the murine bone marrow.^16^

To date, immunotherapeutic DMG studies relied on the use of genetically engineered mouse models (GEMM) or carcinogen-induced models, as these are immunocompetent themselves and can be allografted in syngeneic immunocompetent animals.^17,18^ Unlike PDOX, GEMM can recapitulate tumor initiation and development in animals with a native immune system.^18^ Furthermore, the conditional or inducible systems that have been developed, such as RCAS/Tv-a or Cre-LoxP systems, allow for a target gene to be edited in a tissue-specific and/or time-dependent way.^18^ Nonetheless, GEMM developed to date often do not accurately reflect the molecular background of DMG, lack flexibility due to extensive breeding schemes or develop tumors in inappropriate locations in the brain.^19–22^ Likewise, carcinogen-induced models, such as the commonly used GL261 glioma model, are developed from cerebral cortical tumors, heavily passaged in culture, and generally considered to model adult glioblastoma.^18^ As a result, these models are histologically and genetically distinct from human DMG and are therefore not suitable to model DMG growth or predict therapeutic response.

In this study, we describe a rapid and reproducible platform to generate immunocompetent syngeneic allograft DMG models that adequately recapitulate the native anatomical location and histopathologic features of their human counterparts. We achieved this by transfecting combinations of mutations identified in DMG patients in the developing embryonic brainstem by IUE, creating cell lines from the resulting primary DMG tumors, and subsequently orthotopically injecting these murine DMG cell lines as allografts in syngeneic mice. As described previously, the IUE platform provides a system to manipulate expression of multiple genes simultaneously within the intact developing CNS, thereby creating spontaneously arising tumors in a spatially and temporally defined manner.^24^ Furthermore, these mutations are introduced onto a controlled, isogenic background, which allows for accurate studies into the effect of different mutations on DMG pathogenesis. In previous studies, we showed that these IUE-based brainstem tumors are similar to their human counterparts at the transcriptomic level.^24,41^ Here, we further demonstrate that these IUE-transformed murine tumor cells recapitulate patient-derived cell sensitivity to Panobinostat, one of the most extensively studied compounds in (pre)clinical DMG studies. We have also recently shown that the vasculature of IUE-based DMG mouse models closely resembles the minimally disrupted blood-brain barrier of patient-derived xenograft models, indicating that the models developed in this study are suitable surrogates for testing drug penetration and efficacy of other therapeutics.^42^ Unlike xenografts, the immune competent status of these primary IUE and secondary allograft models will allow for promising novel (immuno)therapeutic strategies, e.g., oncolytic viruses, CAR T-cell therapy, and checkpoint inhibitors, to be tested in an environment that better reflects human disease.

The development and sharing of patient-derived DMG cell lines has fueled advancements in the field over the past decade. This in part due to the ability of most labs to propagate these cells in vitro and then apply lab-specific expertise to answer hypotheses-based questions. Taking a cue from the success of these shared models, we reasoned that creating a syngeneic implant model would build on primary IUE models by providing a tool that is better suitable for large-scale therapeutic studies and more accessible. Notably, one interesting difference between primary IUE vs. secondary orthotopic allograft models is that while K27M histone mutations accelerate tumor formation in IUE mouse models, histone 3 wildtype and K27M-mutants produce secondary tumors at similar rates. In fact, we consistently note that histone 3 wildtype tumor cells grow quicker than K27M cells both in vitro and in vivo, even though these cell lines retain expression of the K27M-mutant and loss of H3K27me2/3. This would indicate K27M mutations are more important for driving DMG initiation rather than tumor proliferation, possibly through its suggested role in inhibiting differentiation programs.^43–46^ Future work to increase the genetic diversity of available allograft mouse models will further enhance the utility of this system to complement the growing catalog of PDOX models. This could include models that substitute PPM1D mutations for loss of TP53 function^41^ or expression of wild-type PDGFRA, mutant FGFR1 or other MAPK pathway mutations^47^ to replace the use of constitutively active Pdgfra-D842V mutations.

Until recently, the TIME had been poorly studied in DMG, which is of vital importance for the rational design of immunotherapy strategies. Much of our knowledge on the immunophenotype of DMG has been obtained through immunohistochemical analyses of biopsy or autopsy tissues, which has consistently shown that DMG tumors are characterized by substantial infiltration of tolerogenic bone marrow-derived macrophages and tissue-resident microglia but very limited T- or NK cells.^10,11^ Furthermore, these tumors do not appear to express the chemokines or cytokines required to recruit these cells, nor do they express significant amounts of immunosuppressive factors, resulting in an immunologically inert microenvironment that may considerably limit the efficacy of various immunotherapeutic compounds.^10,11^ This is in contrast with pediatric low-grade gliomas, cortical gliomas, and adult glioblastomas, which demonstrate more extensive lymphoid cell infiltration compared to adjacent brain tissues, and express high levels of both pro- and anti-inflammatory factors, emphasizing the significance of using the appropriate disease models for DMG research.^11^ While these initial studies comprehensively highlight the unique TIME of DMG, advanced knowledge about immune cell subpopulations that modulate DMG progression is urgently needed. In our syngeneic implant model, we observed a high number of myeloid cells and minimal presence of T-lymphocytes and NK cells, corresponding to previous reports and validating our model as being representative of the human disease. As such, this model represents a valuable tool to further investigate the specific phenotype and (pro- or anti-tumor) function of these DMG-associated myeloid cells and their potential for therapeutic targeting. Moreover, our model enables the investigation of DMG subtype-specific differences to determine how the molecular signature or tumor location dictates the immune landscape, allowing us to better predict outcomes of immunotherapeutic strategies in clinical trials.

Altogether, the method described in this study provides a flexible and reproducible platform to generate immunocompetent murine DMG models that closely recapitulate the histopathological features, spatiotemporal characteristics, and microenvironment composition of their human counterparts. As such, these tools, and the general platform used to generate them, will be valuable for future preclinical studies.

## Supplementary Material

vdac079_suppl_Supplementary_Material_S1

vdac079_suppl_Supplementary_Material_S2
