# Corrigendum to: Generation of immunocompetent syngeneic allograft mouse models for pediatric diffuse midline glioma

**DOI:** 10.1093/noajnl/vdad072

**Published:** 2023-06-10

This is a Corrigendum to: Aimée du Chatinier and others, Generation of immunocompetent syngeneic allograft mouse models for pediatric diffuse midline glioma, Neuro-Oncology Advances, Volume 4, Issue 1, January-December 2022, vdac079, https://doi.org/10.1093/noajnl/vdac079

In the originally published version of this manuscript, there was a duplication within panel A of Figure 4. This should read:


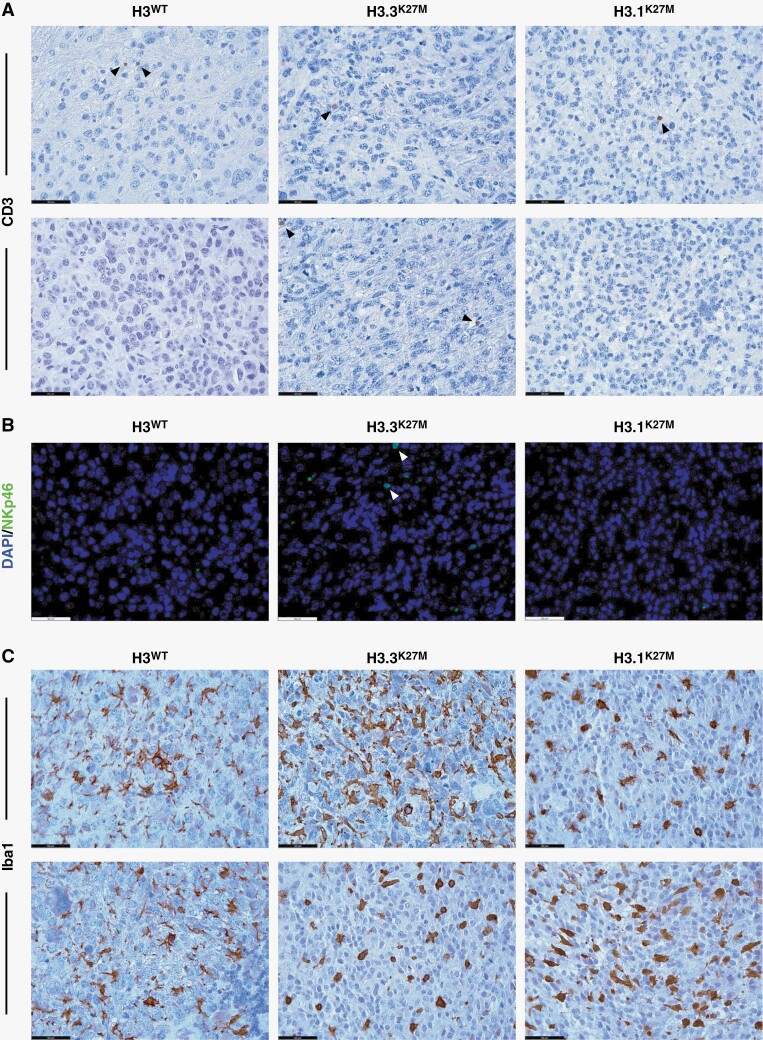


instead of:


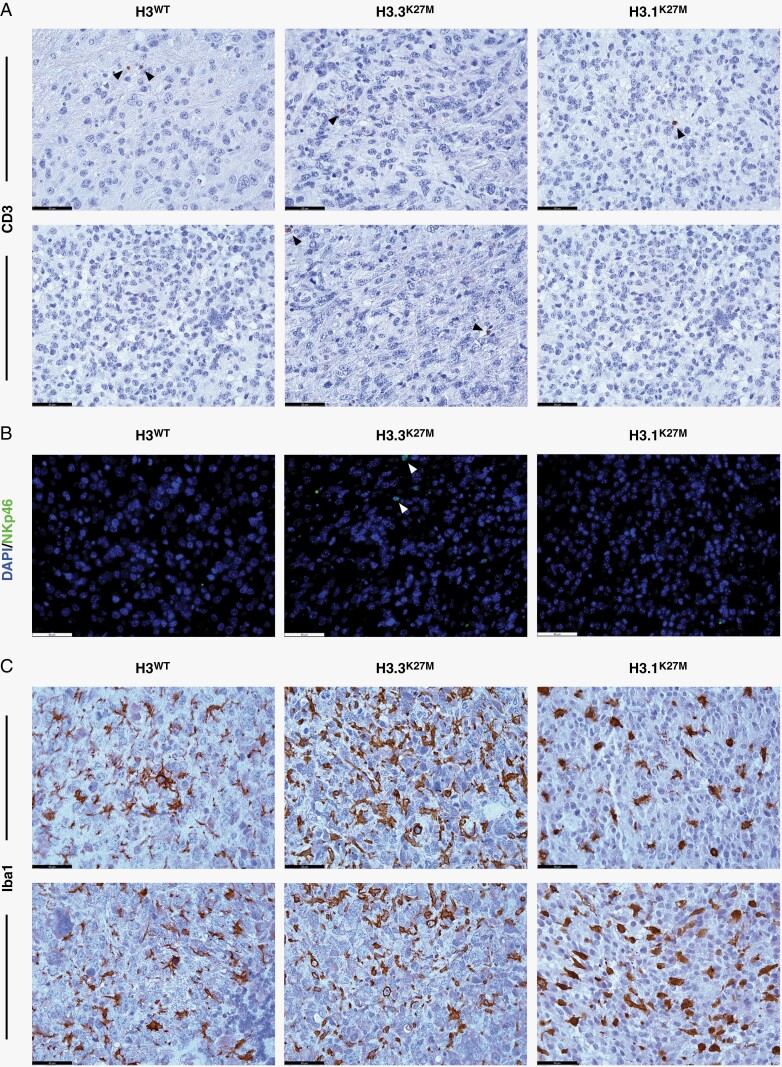


This error has been corrected in the article.

