# Supplementary material for: Generation of immunocompetent syngeneic allograft mouse models for pediatric diffuse midline glioma
Source: Neurooncol Adv. 2022 May 24;4(1):vdac079. doi: 10.1093/noajnl/vdac079 (PMC9210310; doi:10.1093/noajnl/vdac079)
Supplement: vdac079_suppl_Supplementary_Material_S1 [file vdac079_suppl_supplementary_material_s1.docx]

**Supplementary Material**

***In vivo* studies**

As described previously^1^, defined combinations (Supplementary Table 1) of stably integrating PiggyBac DNA plasmids and complementary PiggyBac transposase (pCAG-PBase) were concentrated by ethanol precipitation (1µg/µL final concentration for each plasmid), injected into the fourth ventricle of timed pregnant C57BL/6 (Charles River) embryos at embryonic day 13.5, and electroporated toward the brainstem. Following birth, electroporated offspring was monitored for development of neurologic symptoms related to tumor burden. Upon development of neurological symptoms mice were euthanised and cell lines were derived from individual GFP-positive tumor tissue that was microdissected away from normal brain tissue and enzymatically dissociated into single cell suspensions (described below for CyTOF procedure). Cells were put into culture conditions as described previously.^2^ PiggyBac plasmids used include: PBCAG-H3f3a^WT^-Ires-eGFP, PBCAG-H3f3a^K27M^-Ires-eGFP, PBCAG-DNp53-Ires-Luciferase, PBCAG-Pdgfra^D842V^-Ires-eGFP, PBCAG-Hist1b3b^K27M^-Ires-eGFP, and PBCAG-Acvr1^G328V^-Ires-eGFP. All IUE procedures were performed at the University of Cincinnati.

Secondary syngeneic allograft DMG tumors were generated by stereotactically injecting murine DMG cell lines (Supplementary Table 1) into 6 to 8-week-old female C57BL/6 mice (Charles River) at a final concentration of 100,000 cells/μL (n=3 implants per cell line). The stereotactic coordinates that were used to inject into the pons were -0.8mm laterally, -1mm caudally and -4.5mm ventrally from the lambda. Cells were injected using a volume of 5μL at a flow rate of 2μL/min to keep neurological side effects of the procedure to a minimum and prevent potential backflow of cells. Mice were inspected daily during the initial week after injection, followed by bi-weekly inspection for the remainder of the experiment. Upon reaching their humane endpoint (severe neurological symptoms or >20% loss of total body weight) mice were euthanised by 20% Euthasol® (AST Farma). Brains were extracted, fixed in 4% paraformaldehyde (PFA) for 48h, and embedded in paraffin.

**Immunohistochemistry (IHC) and immunofluorescence (IF)**

For IHC, the following primary antibodies were used: Ki67 (1:500, #MA5-14520, ThermoFisher Scientific), H3K27M (1:500, #ABE419, Millipore), H3K27me2/3 (1:500, #39535, Active Motif), Iba1 (1:200, #AB48004, Abcam), CD3 (mouse) (1:500, #14-0032-85, ThermoFisher Scientific), CD3 (human) (1:100, #A0452, DAKO), and IL13Rα2 (1:300, #BS-2461R, ThermoFisher Scientific). For IF, the following primary antibodies were used: Gfap (1:200, #12389, Cell Signaling Technology), Olig2 (1:200, #AB9610, Sigma Aldrich), and NKp46 (1:200, #PA5-102860, ThermoFisher Scientific), CD45 (1:500, #M0701, DAKO), and CD57 (1:200, #157M-97, Cell Marque-Sigma Aldrich). Images were captured using a Leica DM6 or DMi8 microscope (Leica Microsystems) operated by Leica Application Suite X (LAS X) software.

**Cytometry by Time of Flight (CyTOF) mass cytometry**

Tumor samples from individual IUE DMG mouse models were collected and processed as follows: Mice displaying neurological symptoms related to tumor burden were deeply anesthetized prior to transcardial perfusion with cold DPBS (Gibco #14190144). Next, brains were quickly removed, and GFP-positive tumor tissue was microdissected under a fluorescent stereomicroscope. Isolated tumor tissue was then cut into small pieces with scalpels and transferred into an enzyme cocktail consisting of Collagenase IV (3.2mg/mL, Worthington #LS004209), DNAse1 (1mg/mL, Worthington #LS002007) and Soybean Trypsin Inhibitor (2mg/mL, Worthington #LS003587) dissolved in DPBS. Following incubation in this enzyme mixture at 37˚C, tumor tissue was triturated, washed in DPBS and passed through a 70µm mesh cell strainer. Residual erythrocytes were lysed with ACK buffer (Gibco #A1049201) for 5min followed by quenching with DPBS. The cell suspension was then incubated with myelin removal beads (Miltenyi #130-096-731) and applied to magnetic bead columns (Miltenyi #130-042-201) to remove excess myelin and debris. Purified cell suspensions were incubated with cisplatin (NatPt, 5µM, Enzo #ALX-400-040) for 5min at room temperature and then washed with CSM buffer (DPBS + 0.5% BSA). Next, cells were fixed in a solution of 5mL CSM buffer + 500µL 16% PFA (EMS #15710-S) for 10min at room temperature. Following washes in CSM buffer cells were resuspended in CSM buffer + 10% DMSO and stored at -80˚C. Subsequent antibody conjugation, validation, and staining procedures were performed as described previously.^3^

*CyTOF data acquisition and analysis*

Debarcoded data was first re-scaled by applying ArcSin and pre-processed to exclude beads, doublets, dead cells, and outliers using 193Ir and 140Ce, 193Ir and Event Length, 193Ir and 195Pt, and Gaussian parameters, respectively. The remaining cells were downsampled to 75000 cells per sample to allow fair comparisons between samples. Cells were then gated according to the following strategy: endothelial cells (GFP-CD3-CD31+), GFP+ tumor cells (CD45-GFP+), T cells (CD45+GFP-CD3+, and then CD4+CD8- or CD4-CD8+), microglia (CD45dimGFP-CD3-CD11bHi), macrophages (CD45HiGFP-CD3-CD11bHiF4-80+) and dendritic cells (Cd45HiGFP-CD3-CD11b-F4-80-CD11c+MHCII+). Median mass intensity for different markers was calculated per population. The aforementioned populations were overlaid on to an opt-SNE map that was computed with a perplexity of 30, a Theta of 0.5, and a verbosity of 25 using the following features to build the optSNE variables: CD45, B220, CD11a, Ly-6G, cPARP, CD38, CD49b, CD11c, GFP, CD27, CD206, PD-L1, CD103, CTLA-4, PDCA-1, Ly-6C, Ki67, CD11b, Rb, CD8a, CD4, CD3e, PD-1, CD31, MHC-I, T-bet, Flt3, CD62L, ICOS, CD69, CD492, FoxP3, CD25, F4-80, CSF-1R, CCR2, KLRG1, CCR4, CCR7, CD44, CD90, and MHC-II. OMIQ software was used for data analysis.

**References**

**1.** Patel SK, Hartley RM, Wei X, et al. Generation of diffuse intrinsic pontine glioma mouse models by brainstem-targeted in utero electroporation. *Neuro Oncol.* 2020; 22(3):381-392.

**2.** Meel MH, Sewing ACP, Waranecki P, et al. Culture methods of diffuse intrinsic pontine glioma cells determine response to targeted therapies. *Experimental cell research.* 2017; 360(2):397-403.

**3.** Simonds EF, Lu ED, Badillo O, et al. Deep immune profiling reveals targetable mechanisms of immune evasion in immune checkpoint inhibitor-refractory glioblastoma. *J Immunother Cancer.* 2021; 9(6).
