# Supplementary material for: Generation of immunocompetent syngeneic allograft mouse models for pediatric diffuse midline glioma
Source: Neurooncol Adv. 2022 May 24;4(1):vdac079. doi: 10.1093/noajnl/vdac079 (PMC9210310; doi:10.1093/noajnl/vdac079)
Supplement: vdac079_suppl_Supplementary_Material_S2 [file vdac079_suppl_supplementary_material_s2.docx]

**Supplementary Tables and Figures**

**Supplementary Table 1: Overview genetic background murine DMG models**

| **DMG tumor model** | **Cell line ID** | **IUE-transduced PiggyBac plasmids** |
| --- | --- | --- |
| H3^WT^ | UC-BL6-D1  UC-BL6-D3 | H3f3a^WT^  DNp53*^a^*  Pdgfra^D842V^ |
| H3.3^K27M^ | UC-BL6-B1  UC-BL6-B7 | H3f3a^K27M^  DNp53  Pdgfra^D842V^ |
| H3.1^K27M^ | UC-BL6-C1  UC-BL6-C2  UC-BL6-C7 | Hist1h3b^K27M^  DNp53  Pdgfra^D842V^  Acvr1^G328V^ |

*a* DNp53: dominant negative Trp53


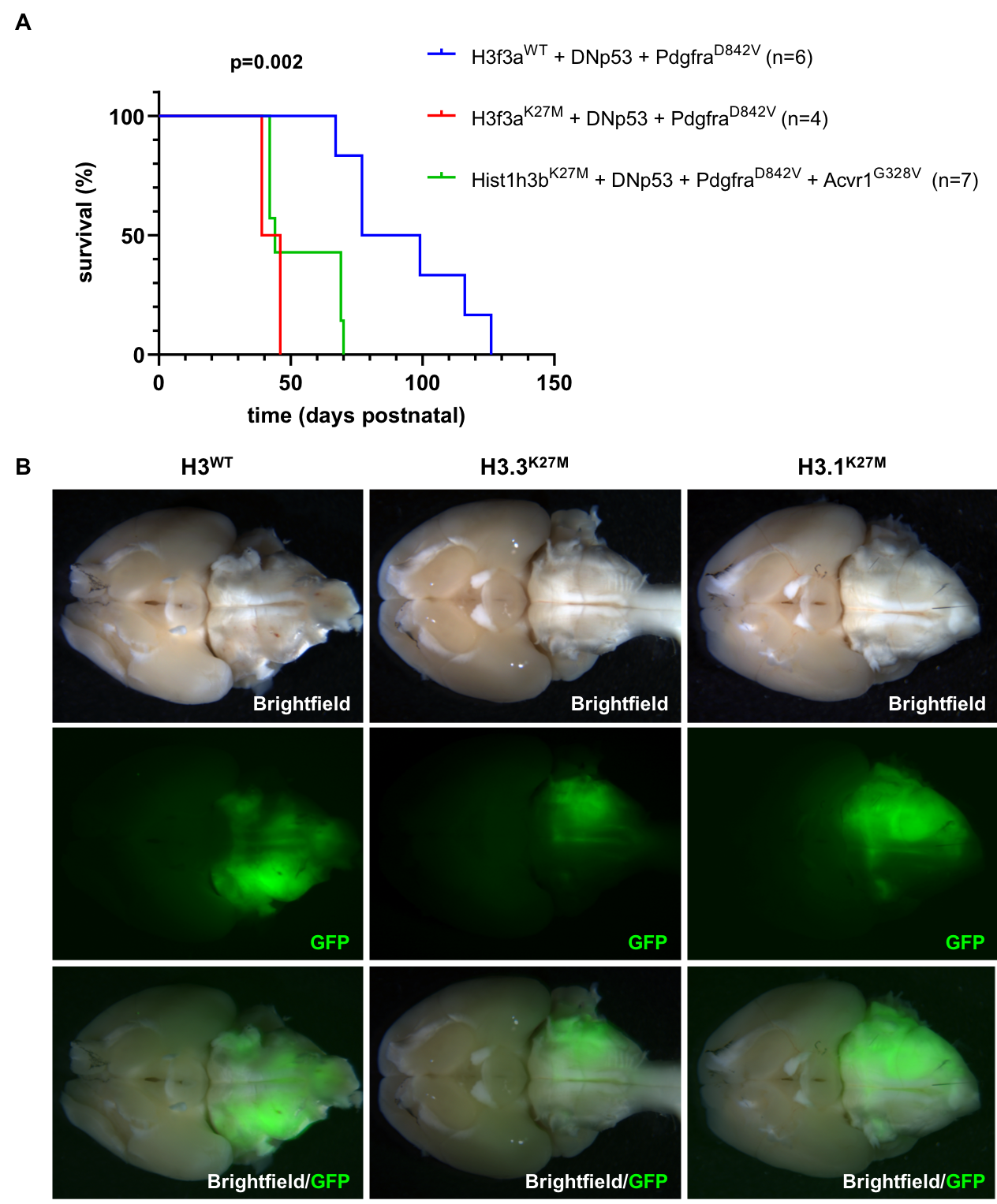


**Supplementary Fig. S1**

(A) Kaplan-Meier curves representing survival of C57BL/6 mice with primary murine DMG tumors generated by brainstem targeted IUE of PiggyBac DNA plasmids. (B) Representative ventral whole brain brightfield and GFP images demonstrating the location of GFP-positive H3^WT^, H3.3^K27M^, and H3.1^K27M^ tumors generated by IUE.

**
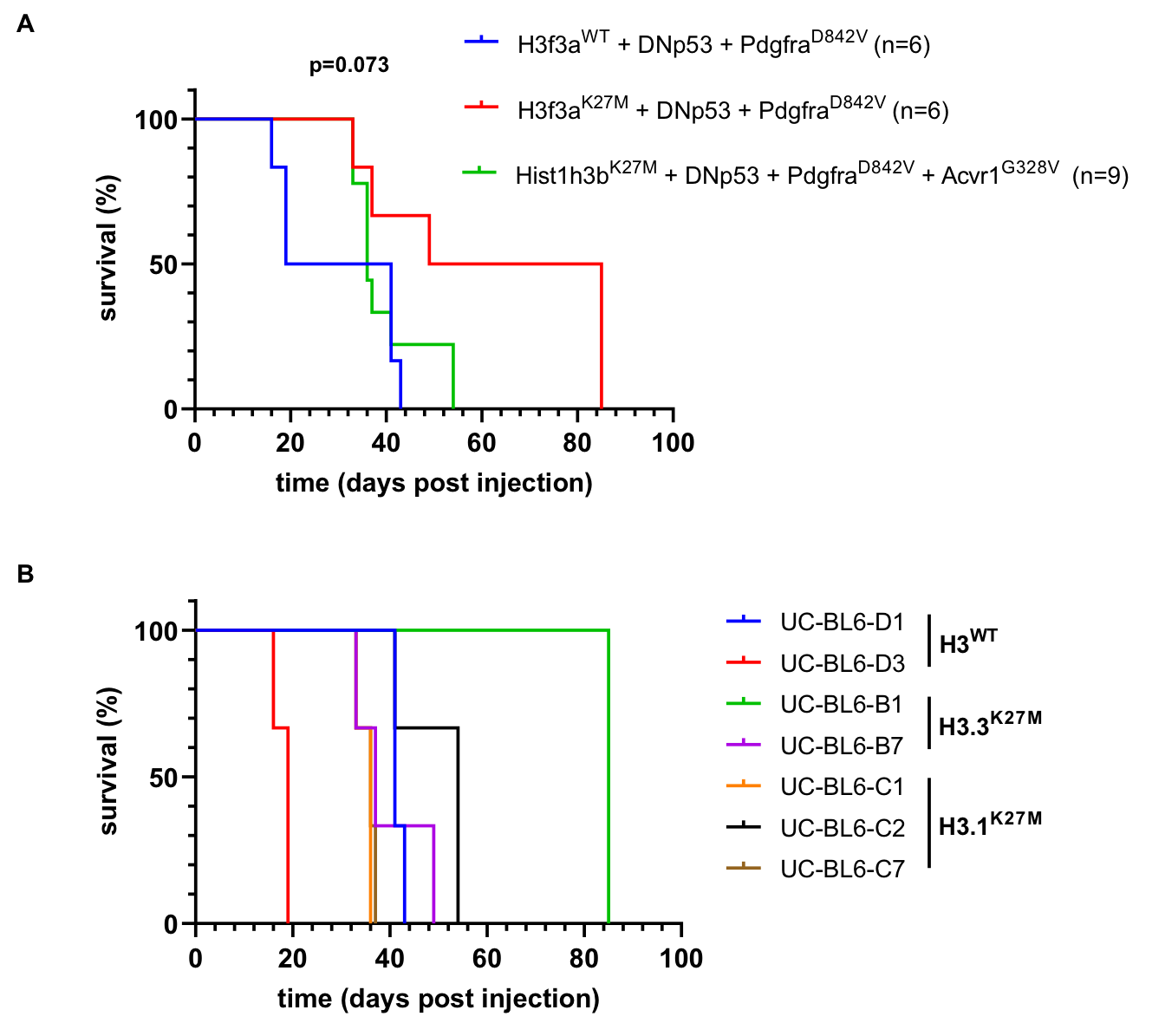
**

**Supplementary Fig. S2**

(A) Kaplan-Meier curves representing survival of C57BL/6 mice orthotopically injected with IUE-transformed murine DMG cells (i.e., syngeneic allograft DMG models) across genetic conditions. (B) Kaplan-Meier curves representing survival of syngeneic allograft DMG models per established cell line (n=3 implants per cell line).

**
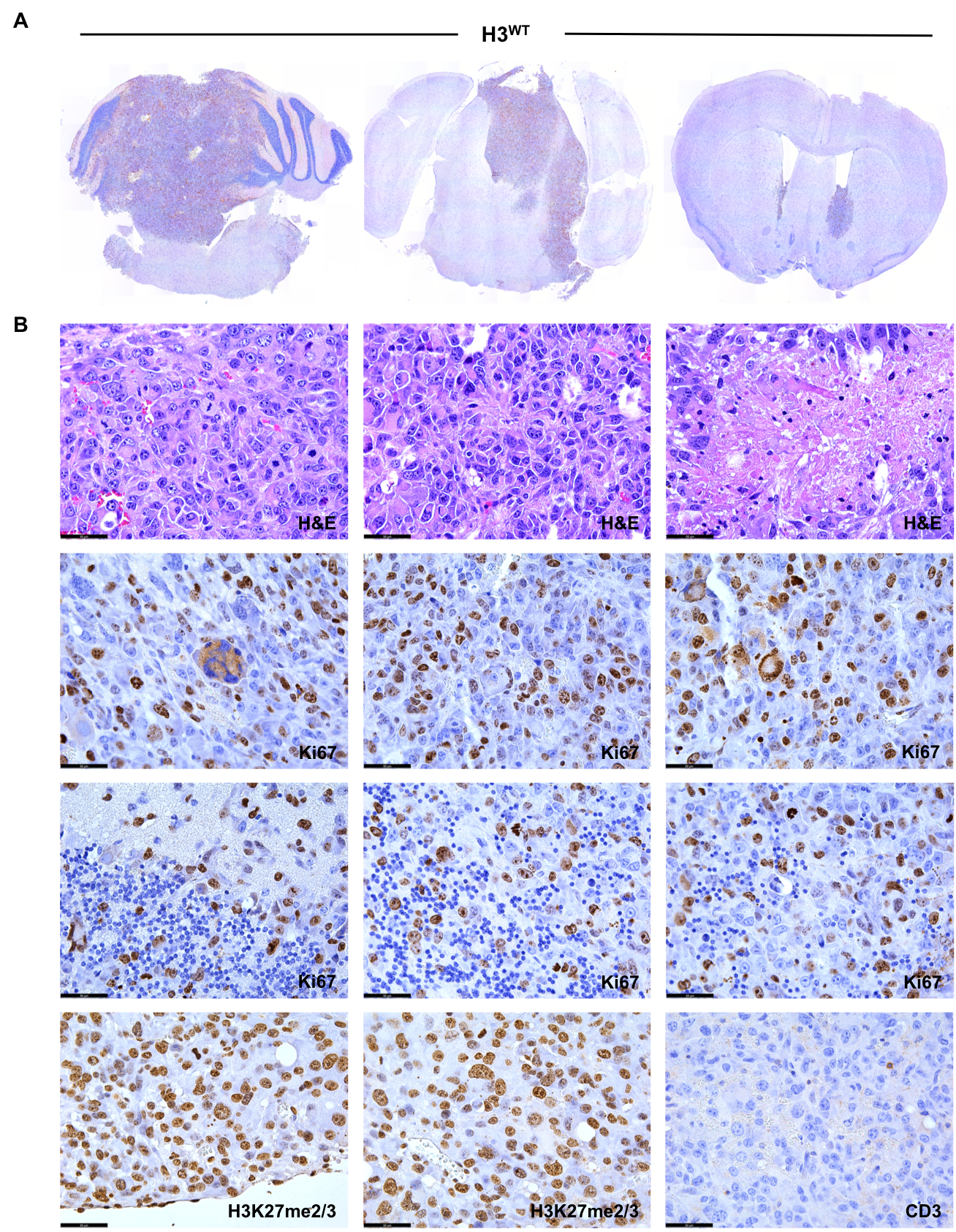
**

**Supplementary Fig. S3**

(A) Representative coronal sections of Ki67 immunohistochemical staining (in brown) of H3^WT^ DMG allograft brain sections, showing the tumor spread throughout the midbrain, into the cerebellum, and alongside the ventricles into the cerebral cortex. (B) Supplementary images (400x) of immunohistochemical staining of H3^WT^ DMG allograft tumor sections showing (from left to right) the tumor core (H&E, Ki67), necrotic areas (H&E), giant cells (Ki67), infiltration into the cerebellum (Ki67), histone 3 lysine 27 di- and trimethylation conservation in some cells and loss in others (H3K27me2/3), and CD3-positive T-lymphocytes identified around hemorrhagic areas. Scale bars = 50μm.


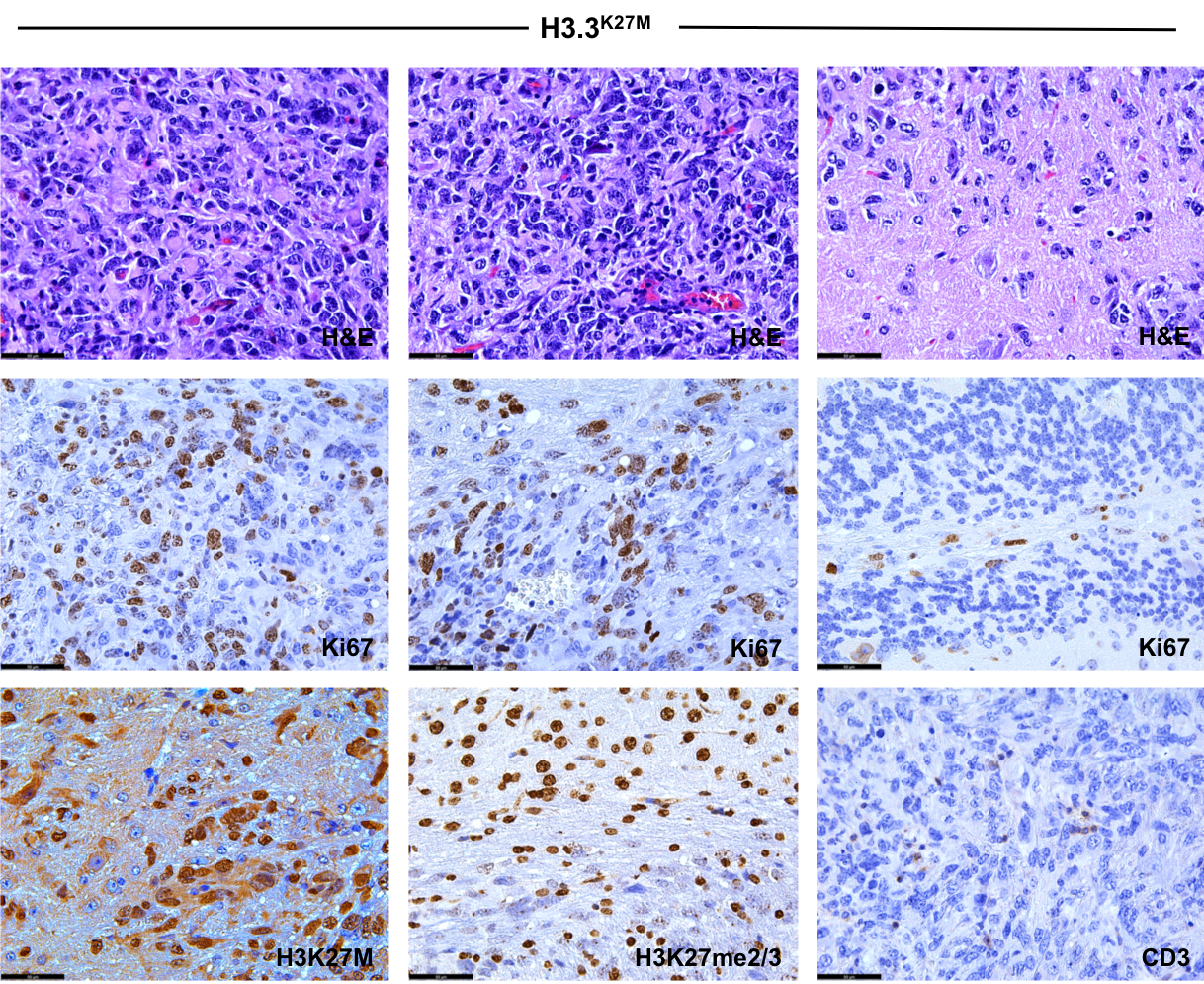


**Supplementary Fig. S4**

Supplementary images (400x) of immunohistochemical staining of H3.3^K27M^ DMG allograft tumor sections showing (from left to right) the tumor core and infiltrative areas (H&E, Ki67), infiltration into the cerebellum (Ki67), immunopositive staining for the mutant histone 3 protein in tumor nuclei in the infiltrative areas (H3K27M), histone 3 lysine 27 di- and trimethylation loss in tumor areas adjacent to healthy brain tissue (H3K27me2/3), and a rare hotspot of CD3-positive T-lymphocytes. Scale bars = 50μm.


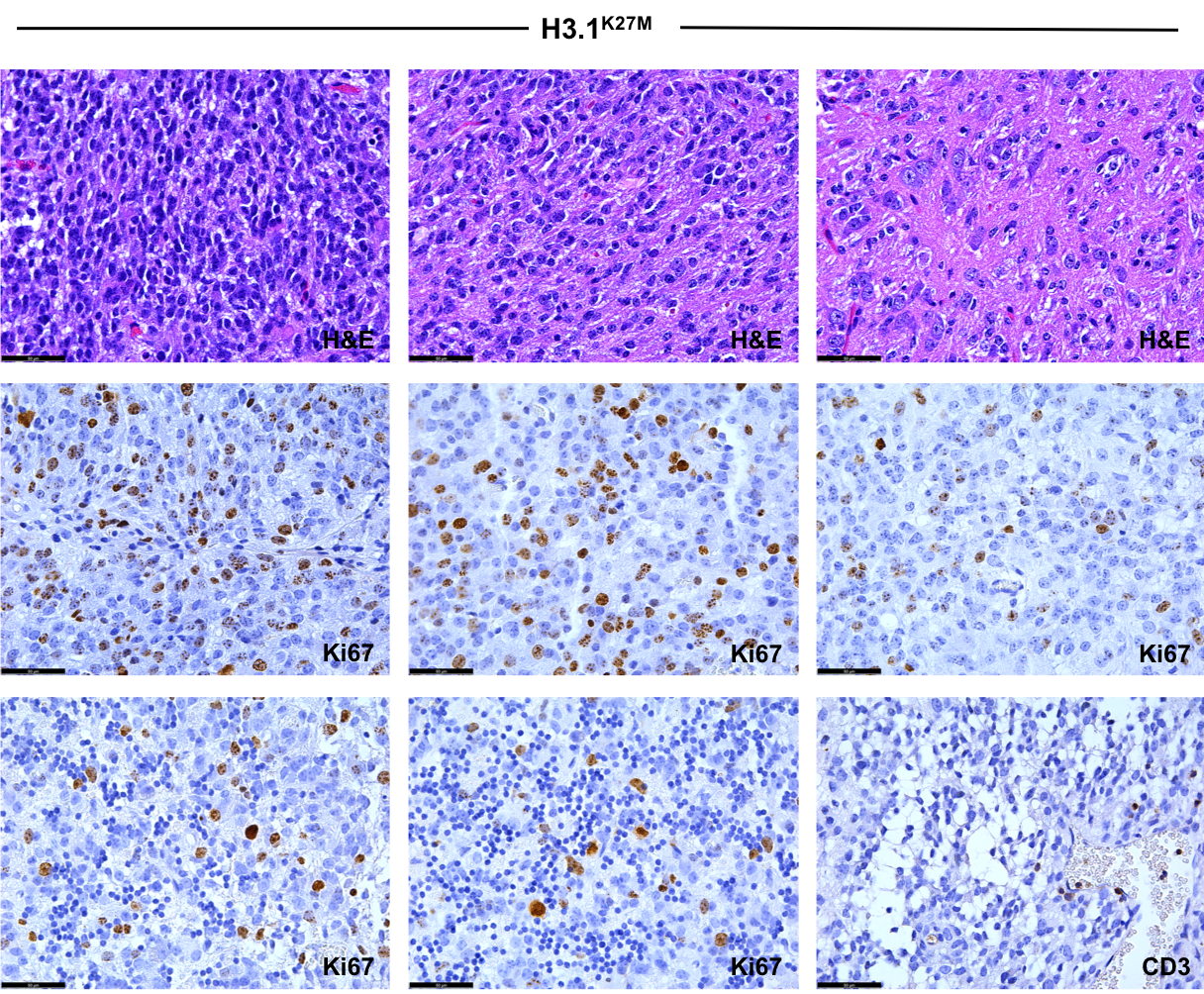


**Supplementary Fig. S5**

Supplementary images (400x) of immunohistochemical staining of H3.1^K27M^ DMG allograft tumor sections showing the tumor core and infiltrative areas (H&E), intra-tumoral heterogeneity with respect to cellular density of proliferative Ki67-positive cells (Ki67), infiltration into the cerebellum (Ki67), and a rare hotspot of CD3-positive T-lymphocytes in and around a blood vessel. Scale bars = 50μm.


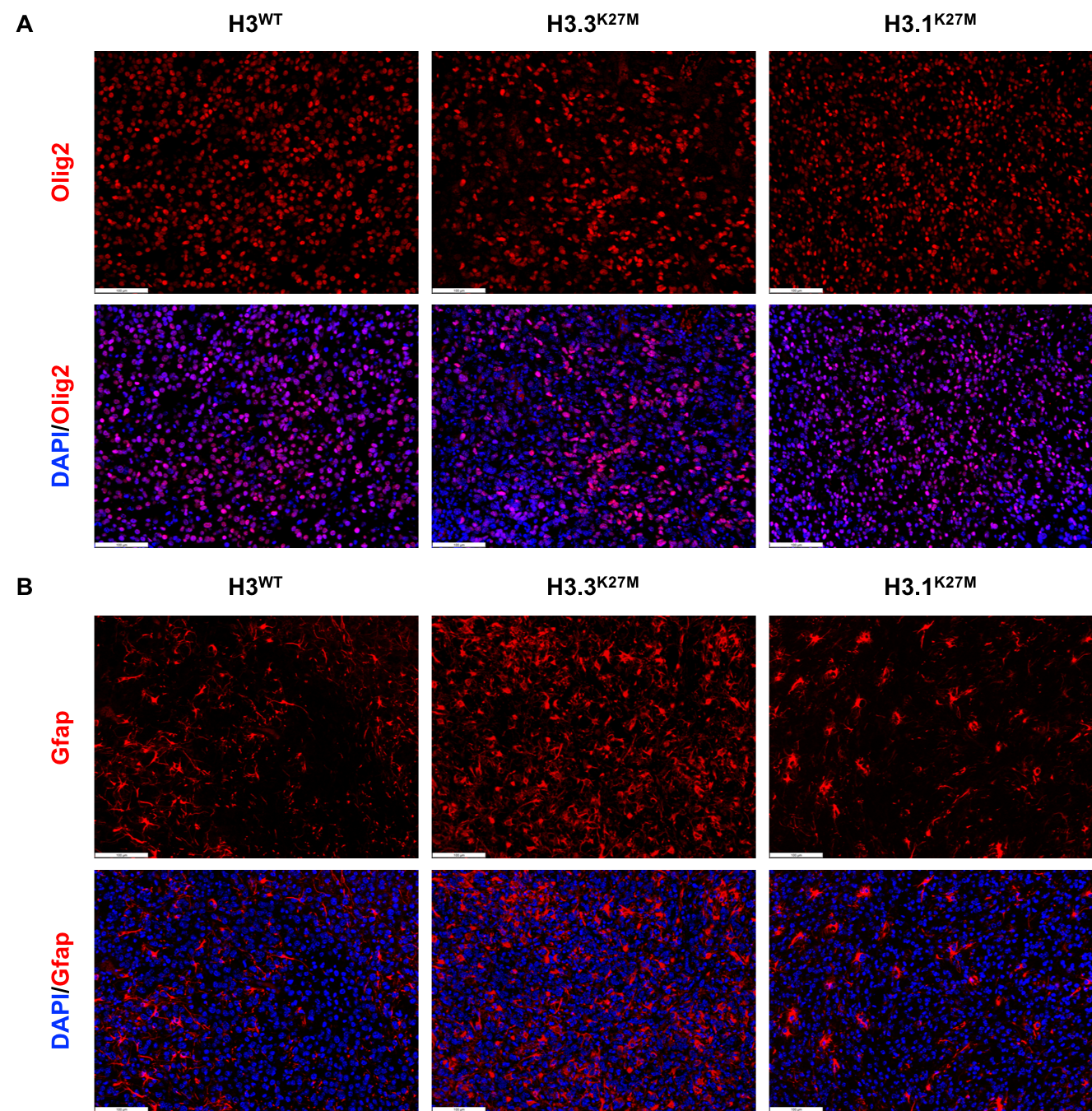
**Supplementary Fig. S6**

Representative immunofluorescent images (200x) of DMG allograft tumors across genetic conditions co-stained for DAPI (blue) and (A) oligodendrocyte transcription factor 2 (Olig2) (red) or (B) glial fibrillary acidic protein (Gfap) (red). Scale bars = 100μm.


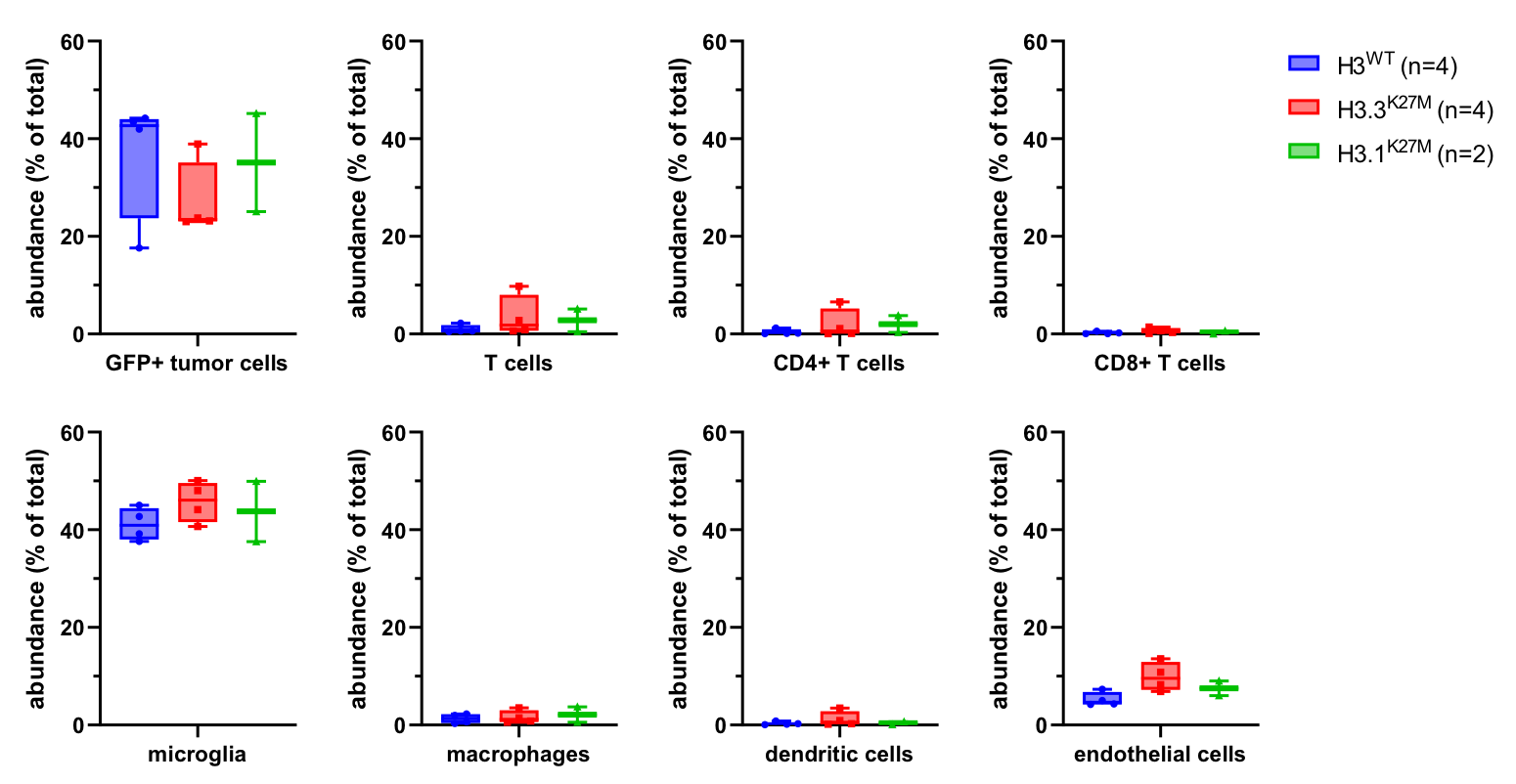


**Supplementary Fig. S7**

Box and whisker plot representing relative abundance of indicated cell populations in whole primary IUE brains across genetic conditions (based on CyTOF mass cytometry analysis, Fig. 3).

**
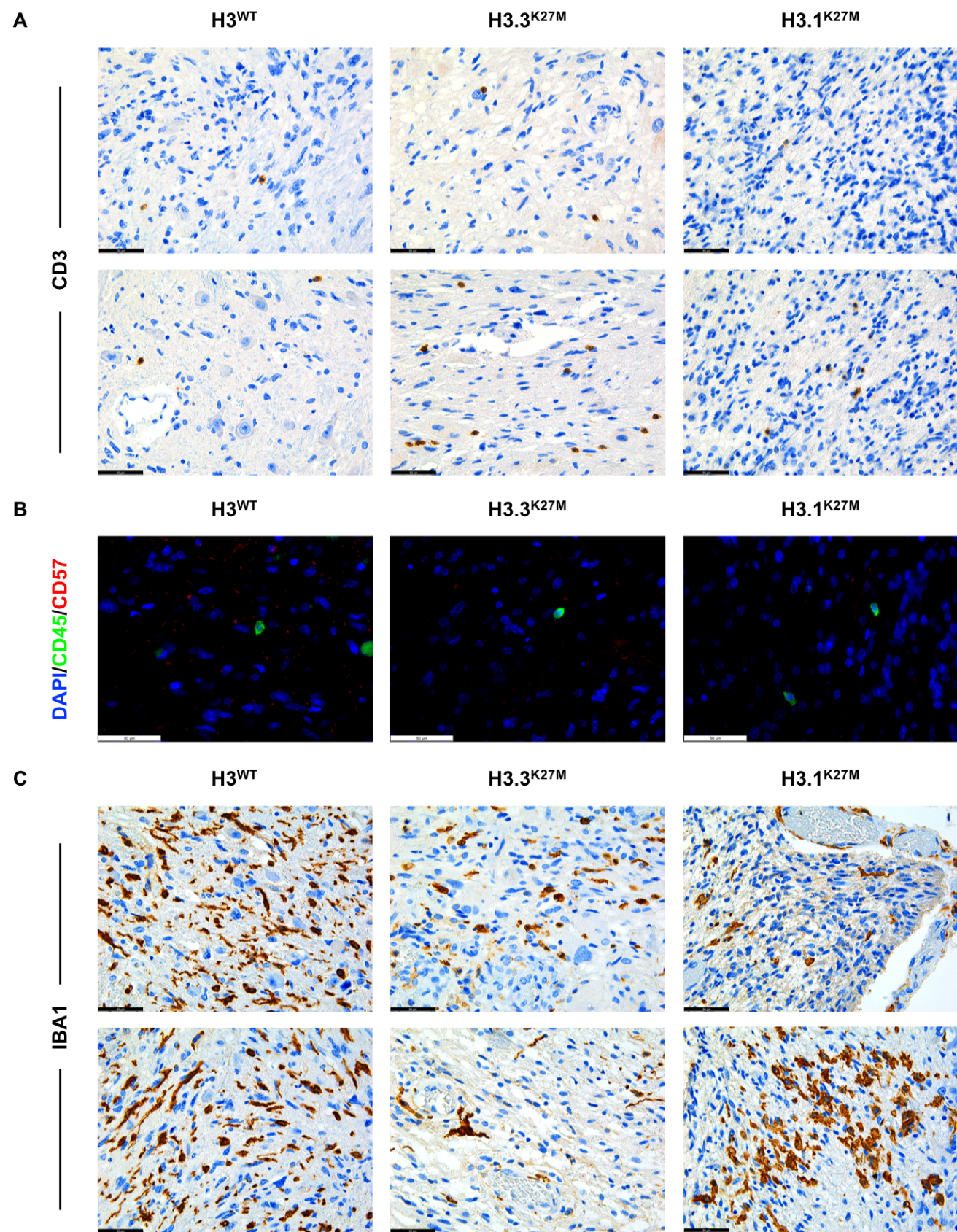
**

**Supplementary Fig. S8**

(A) Representative images (400x) of CD3 immunohistochemical staining (in brown; marker for T-lymphocytes) of patient derived DMG tumors across genetic conditions. (B) Representative immunofluorescent (400x) images of patient derived DMG tumor sections co-stained for DAPI (blue), CD45 (green; marker for inflammatory cells), and CD57 (red; marker for NK cells). (C) Representative images (400x) of Iba1 immunohistochemical staining (in brown; marker for microglia and macrophages) of patient derived DMG tumor sections, showing the intra-tumoral heterogeneity with respect to microglia/macrophage density and phenotype. Scale bars = 50μm.


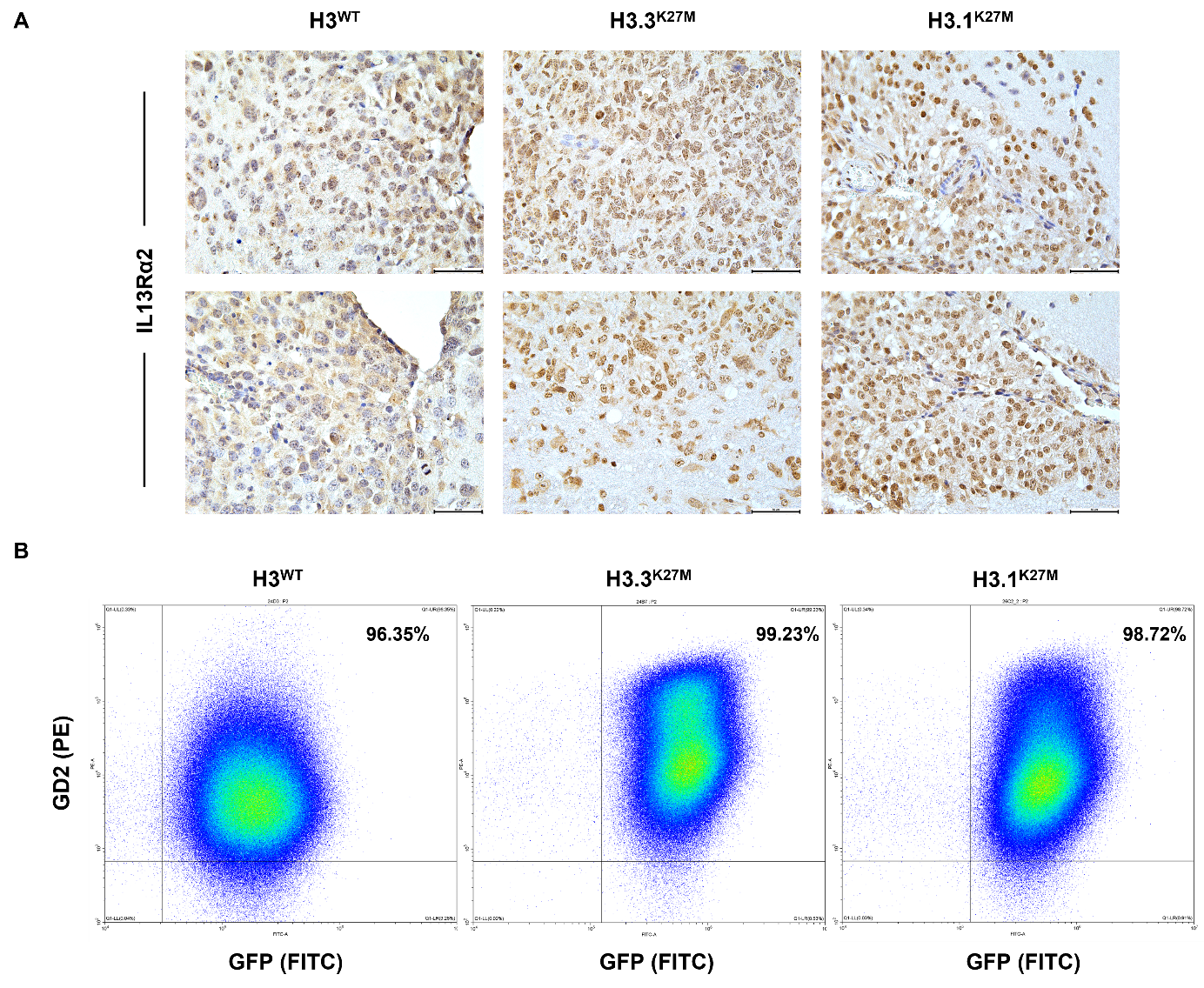
**Supplementary Fig. S9**

(A) Representative images (400x) of immunohistochemical staining (in brown) for IL13Rα2 of DMG allograft tumor sections. Scale bars = 50μm. (B) Flow cytometric analysis of GFP-positive murine DMG tumor cells stained for GD2 (PE-labeled).
